# Antigenic-Specificity and Cytokine Profile of the T-Cell Response to Human Cytomegalovirus in Transplant Recipients

**DOI:** 10.3390/pathogens15010053

**Published:** 2026-01-05

**Authors:** Federica Zavaglio, Paola Zelini, Asja Cera, Piera d’Angelo, Marilena Gregorini, Teresa Rampino, Lucia Del Frate, Federica Meloni, Oscar Borsani, Carlo Pellegrini, Fausto Baldanti, Daniele Lilleri

**Affiliations:** 1 Microbiology and Virology Unit, Fondazione IRCCS Policlinico San Matteo, 27100 Pavia, Italy; f.zavaglio@smatteo.pv.it (F.Z.); p.dangelo@smatteo.pv.it (P.d.); f.baldanti@smatteo.pv.it (F.B.); d.lilleri@smatteo.pv.it (D.L.); 2 Institute for Research in Biomedicine, 6500 Bellinzona, Switzerland; asja.cera@icloud.com; 3 Unit of Nephrology, Dialysis and Transplantation, Fondazione IRCCS Policlinico San Matteo, 27100 Pavia, Italy; m.gregorini@smatteo.pv.it (M.G.); t.rampino@smatteo.pv.it (T.R.); 4 Department of Internal Medicine and Therapeutics, University of Pavia, 27100 Pavia, Italy; 5 Transplant Centre Unit, Fondazione IRCCS Policlinico San Matteo, 27100 Pavia, Italy; lucia.delfrate01@universitadipavia.it; 6 Respiratory Diseases DCTV, University of Padova & AOPD, 35122 Padova, Italy; federica.meloni@unipd.it; 7 Department of Molecular Medicine, University of Pavia, 27100 Pavia, Italy; o.borsani@smatteo.pv.it; 8 Department of Hematology Oncology, Fondazione IRCCS Policlinico San Matteo, 27100 Pavia, Italy; 9 Cardiac Surgery, Department of Intensive Medicine, Fondazione IRCCS Policlinico San Matteo, 27100 Pavia, Italy; c.pellegrini@smatteo.pv.it; 10 Department of Clinical, Surgical, Diagnostic and Pediatric Sciences, University of Pavia, 27100 Pavia, Italy

**Keywords:** human cytomegalovirus infection, transplant recipients, antigenic-specific T-cell response, cytokine profile of T-cell response

## Abstract

Human cytomegalovirus (HCMV) infection is a significant complication in transplant recipients. Following HCMV reactivation, the recovery of T-cell responses serves as a key indicator of protection from HCMV disease. This study aimed to assess the HCMV-specific CD4^+^ and CD8^+^ T-cell responses and their cytokine production (IFNγ, TNFα, IL2) against various HCMV proteins (IE-1, pp65, gB, gH/gL/pUL128L) in solid organ transplant recipients (SOTRs) and hematopoietic stem cell transplant recipients (HSCTRs) with active HCMV infection. The cohort consisted of 16 SOTR and 16 HSCTR categorized into two groups: (i) Controllers, who spontaneously controlled the infection, and (ii) Non-Controllers, who required antiviral treatment. T-cell responses were analyzed following stimulation with peptide pools and intracellular cytokine staining. Prior to transplantation, all patients exhibited a significantly higher frequency of CD4^+^ T cells specific to pp65 compared to gH and gL/pUL128L. During the peak of infection, T-cell frequencies across all peptides were similar, but at infection resolution, the frequency of pp65 and gB-specific CD4^+^IFNγ^+^ T cells was significantly higher than gL/pUL128L. Additionally, pp65 and IE-1-specific CD8^+^IFNγ^+^ T-cell responses were significantly greater than those against gH and gL/pUL128L at the resolution of infection. Notably, Controllers exhibited significantly higher frequencies of monofunctional pp65-specific T cells, particularly in CD8^+^ T cells producing IFNγ and TNFα. The response to pp65, especially IFNγ production, may serve as a key marker for identifying patients capable of controlling HCMV infection.

## 1. Introduction

Solid organ transplant recipients (SOTRs) and hematopoietic stem cell transplant recipients (HSCTRs) are at an increased risk of infections due to immunosuppressive therapies required to prevent graft rejection. Among these infections, human cytomegalovirus (HCMV) remains a significant cause of morbidity and mortality, particularly in the early post-transplantation period [1,2,3,4,5,6,7,8]. Due to the immunosuppressive therapy that transplant recipients undergo, these individuals are at risk of reactivating latent HCMV, which may originate from their own body or the transplanted organ. Such reactivations can lead to either disseminated disease, also known as systemic syndrome, or tissue-invasive disease (TID). Currently, two primary strategies are employed to prevent HCMV-related complications: universal prophylaxis and active surveillance with preemptive therapy. Universal prophylaxis involves the administration of antiviral drugs to all transplant recipients for a period of 6–12 months for SOTR and 100 days for HSCTR (although a recent study proposed the extension of letermovir prophylaxis until 200 days in high risk HSCTR) [9], while active surveillance relies on monitoring blood viral loads and initiating antiviral therapy once viral levels exceed predetermined thresholds. However, a subset of patients may not require either prophylactic treatment or active surveillance, as they either do not experience significant HCMV-specific T-cell dysfunction or exhibit early immune reconstitution, enabling effective control of HCMV reactivation [10,11]. HCMV-specific T cells play a critical role in preventing HCMV disease, with both CD4^+^ and CD8^+^ T cells being essential components of the initial specific cellular immune response in HCMV-seropositive transplant recipients. These cells are also involved in the long-term control of HCMV reactivation [12,13,14,15]. In particular, the strength and persistence of HCMV-specific CD4^+^ T-cell response correlate with a reduced incidence of HCMV disease and reactivation [16]. Therefore, understanding the specific T-cell response against HCMV antigens is crucial for developing effective therapeutic and preventive strategies. Studies have observed the presence of a strong HCMV-specific CD4^+^ T-cell response against IE-1, pp65, and gB proteins [17,18,19,20,21]. Moreover, CD4^+^ T-cell response to the viral lysate stimulation has also been described [22,23]. These antigens are key targets for both CD4^+^ and CD8^+^ T-cell response, and the investigation of this response has provided valuable insights into viral control mechanisms and immune evasion strategies. A comprehensive evaluation of the T-cell response against different HCMV antigens (non-structural, tegument, and envelope antigens) in both SOTR and HSCTR is missing.

The aim of this study was to analyze the HCMV-specific CD4^+^ and CD8^+^ T-cell response, along with their cytokine production (IFNγ, TNFα, IL2), against various HCMV proteins in SOTR and HSCTR patients with active infection. We analyzed in parallel the two categories of transplant recipients in order to identify common patterns of immune protection of HCMV infection. In addition to IE-1, pp65, and gB, which are known to be major T-cell targets, the T-cell response against the pentameric complex gH/gL/pUL128L, which is known to elicit a strong neutralizing antibody response, was also investigated.

## 2. Materials and Methods

### 2.1. Study Population

Sixteen HCMV-seropositive HSCTR and sixteen HCMV-seropositive SOTR, including thirteen kidney transplant recipients (KTRs), two heart transplant recipients (HTRs), and one lung transplant recipient (LTR), were enrolled at Fondazione IRCCS Policlinico San Matteo, Pavia, Italy. Clinical and demographic characteristics are included in Table 1. Regarding SOTR, the HCMV serological status was positive in five donors, negative in six donors, and unknown in five donors. For HSCTR, the HCMV serological status was positive in thirteen donors and negative in three donors. After receiving a transplant, patients were monitored for HCMV DNAemia in whole blood [24] once a week for the first eight weeks, then every 15 days until the fourth month, and finally once a month until a year post-transplantation or in the presence of HCMV-related clinical symptoms. HCMV DNA was quantified using in-house real-time PCR performed on blood samples [21], and the lower limit of detection is 90 HCMV DNA copies/mL. Antiviral therapy with ganciclovir (GCV) or valganciclovir (VGCV) was administered pre-emptively, after the detection of 300,000 HCMV DNA copies/mL in whole blood for SOTR [25], and after the detection of 10,000 HCMV DNA copies/mL in whole blood for HSCTR [25], or in case of suspected or diagnosed HCMV disease. All patients were divided in two groups: a group of 15 patients had self-resolving infection and were named Controllers, while the other group of 17 patients were treated for systemic infection with antiviral drugs and were named Non-Controllers (Table 1). The samples at pre-transplant for SOTR were obtained immediately before the procedure, whereas for HSCTR, they were collected prior to the conditioning therapy. All HSCTR received an allogeneic hematopoietic stem cell transplant. The HSCTR characteristics regarding HLA mismatch and GVHD are included in Table 2. The study was approved by the Ethics Committee and the Fondazione IRCCS Policlinico San Matteo Institutional Review Board (Procedure no. 20180034325 and 20170035082) and patients gave written informed consent.

### 2.2. Protein Peptide Pool

To evaluate the antigen-specific T-cell response, the peptide pool representative of IE-1 (JPT, Peptide Technologies, Berlin, Germany), pp65, gB, and gH/gL/pUL128L (15 mers, overlapping by 10 amino acids, all from A&A Labs LLC, San Diego, CA, USA), were used. A peptide pool of human actin (15 mers, overlapping by 10 amino acids, Pepscan, Le-lystad, The Netherlands) was used as a negative control.

### 2.3. Stimulation with HCMV-Specific Peptide Pool

HCMV-specific CD4^+^ and CD8^+^ T cells from peripheral blood mononuclear cells (PBMCs) were stimulated with the HCMV-specific peptide pool from IE-1, pp65, gB, and gH/gL/pUL128L, the peptide pool of human actin [1 µg/mL] in the presence of 0.5 µg/mL co-stimulator molecules, CD28 and CD49d (BD Biosciences, San Jose, CA, USA), and Brefeldin A (Sigma–Aldrich–Merck, Darmstadt, Germany) at a final concentration of 10 µg/mL, for 16–18 h. Cells were seeded in 96-well round bottom plates at a density of 0.5–1 × 10^6^ cells/200 µL culture medium per well. The culture medium was RPMI 1640 (Euroclone, Milan, Italy) supplemented with 100 U/mL penicillin, 100 μg/mL streptomycin (Euroclone), 2 mM L-glutamine (Euroclone), and 10% of heat-inactivated fetal bovine serum (FBS). PBMC were then incubated overnight at 37 °C with 5% CO_2_ [26].

### 2.4. Intracellular Cytokine Staining

Cells were stained for intracellular IFNγ, IL2, and TNFα production [27]. PBMC were washed with PBS 2mM EDTA and stained in PBS 5% FBS with CD8 V500 (BD, Biosciences) for 30 min at 4 °C. Cells were then washed with PBS 5% FBS, fixed, and permeabilized using Citofix/Citoperm (BD Biosciences) for 20 min at 4 °C. Final staining was performed with CD3 PerCP-Cy 5.5, CD4 APC-Cy7, IFN-γ PE-Cy7, IL-2 APC, and TNFα FITC (all from BD, Biosciences) antibodies in a Perm/Wash buffer (BD Biosciences) for 45 min at room temperature [27]. Cells were then washed with a Perm/Wash buffer and resuspended in PBS 1% paraformaldehyde (Sigma–Aldrich–Merck).

Analysis was performed with FACS Canto II flow cytometer using the FACSDiva™ v6.1.3 software (BD Biosciences). Antigen-specific T-cell response was quantified as the sum of all functional subsets (polyfunctional IFNγ^+^TNFα^+^IL2^+^, IFNγ^+^TNFα^+^, IFNγ^+^IL2^+^, TNFα^+^IL2^+^ and monofunctional IFNγ^+^, TNFα^+^, IL2^+^) present after stimulation. Bi- and trifunctionality were assessed using the “and” or “not” logical gating strategies implemented in the FACS DIVA v6.1.3 software. The gating strategy is shown in Supplementary Figure S1. A value ≥ 0.05% of antigen-specific CD4^+^ and CD8^+^ T cells was considered positive. The absolute number of CD3^+^CD4^+^ and CD3^+^CD8^+^ T-cell count was measured in whole blood using Flow Cytometry (BD Multitest^™^ CD3/CD8/CD45/CD4 with BD TruCOUNT^™^ Tubes, BD Biosciences). The number of antigen-specific CD4^+^ and CD8^+^ T cells/μL blood was calculated by multiplying the percentage of HCMV-specific T cells by the corresponding absolute CD4^+^ and CD8^+^ T-cell count. The mono-, bi-, and trifunctional antigen-specific CD4^+^ and CD8^+^ T-cell response was expressed as the percentage of T cells with a determined cytokine profile out of total antigen-specific T cells.

### 2.5. Statistical Analysis

Quantitative variables were shown as median and interquartile range (IQR), while qualitative variables were shown as frequencies or percentages. Antigen-specific CD4^+^ and CD8^+^ T-cell responses were compared using the Friedman test for multiple comparison of paired data. The Mann–Whitney U-test was used to compare antigen-specific CD4^+^ and CD8^+^ T-cell responses in Controllers and Non-Controllers at different time points. The analyses were performed using GraphPad Prism 8.3.0 (GraphPad Software Inc., La Jolla, CA, USA). All the tests were two-tailed and *p* value < 0.05 was considered statistically significant.

## 3. Results

### 3.1. Immunological Monitoring

All thirty-two patients were divided in two groups: 15/32 (46.8%) patients (six HSCT and nine SOTR) had self-resolving infection and were named Controllers while 17/32 (53.1%) patients (ten HSCT and seven SOTR) were treated for systemic infection with antiviral drugs and were named Non-Controllers. HCMV-specific T-cell response against IE-1, pp65, gB, and gH/gL/pUL128L was determined at three time points in the two groups of patients: in pre-transplant, at HCMV DNA peak, a median time of 72 days (IQR 42–132 days after transplant) in Controllers and 52 days (IQR 38–75 days after transplant) in Non-Controllers; and at resolution of HCMV infection, 226 days (IQR 132–297 days after transplant) in Controllers and 206 days (IQR 141–315 days after transplant) in Non-Controllers. No differences in terms of demographic characteristics and type of transplant were observed in the two groups of patients, as reported in Table 1.

### 3.2. Antigen-Specific CD4^+^ and CD8^+^ T-Cell Response

**Figure 1:**
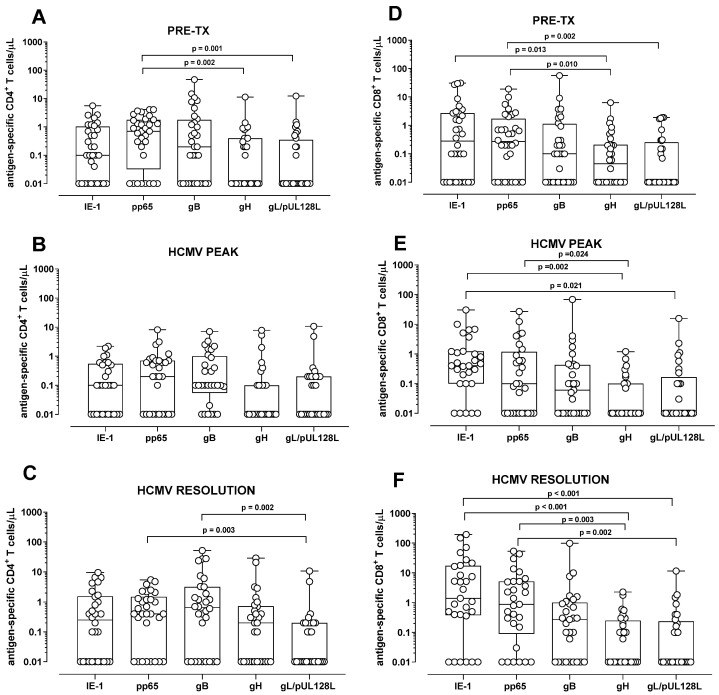
Antigen-specific CD4^+^ and CD8^+^ T-cell response in solid organ transplant recipients and in hematopoietic stem cell recipients after stimulation with IE-1, pp65, gB, gH, and gL/pUL128L at pre-transplant (pre-tx), at HCMV DNA peak, and at the resolution of HCMV infection. The HCMV-specific CD4^+^ T-cell response was quantified as the sum of all functional subsets (polyfunctional IFNγ^+^TNFα^+^IL2^+^, IFNγ^+^TNFα^+^, IFN^+^IL2^+^, TNFα^+^IL2^+^, and monofunctional IFNγ^+^, TNFα^+^, IL2^+^) present after stimulation. (**A**) Antigen-specific CD4^+^ T-cell response at pre-tx. (**B**) Antigen-specific CD4^+^ T-cell response at HCMV DNA peak. (**C**) Antigen-specific CD4^+^ T-cell response at resolution of HCMV infection. (**D**) Antigen-specific CD8^+^ T-cell response at pre-tx. (**E**) Antigen-specific CD8^+^ T-cell response at HCMV DNA peak. (**F**) Antigen-specific CD8^+^ T-cell response at resolution of HCMV infection. The analysis were performed using the Friedman test for paired measures.

In all patients, the HCMV-specific CD4^+^ T-cell response was significantly higher against pp65 compared to gH and gL/pUL128L response (*p* = 0.002 and *p* = 0.001, respectively) at pre-transplant (Figure 1A). For the other peptide pools, no differences were observed (Figure 1A). Instead, at the HCMV DNA peak, no difference in the HCMV-specific CD4^+^ T-cell response was detected among the different peptide pools (Figure 1B). At the resolution of HCMV infection, the CD4^+^ T-cell response was higher against pp65 and gB compared to gL/pUL128L response (*p* = 0.002 and *p* = 0.003, respectively), while no difference was observed among the other peptide pools (Figure 1C). The HCMV-specific CD8^+^ T-cell response was higher against IE-1 and pp65 than gH and gL/pUL128L (*p* = 0.013, *p* = 0.010, and *p* = 0.002, respectively) at pre-transplant (Figure 1D), but no difference was observed for the other peptide pools. At HCMV DNA peak, IE-1-specific CD8+ T-cell response was higher than gH and gL/pUL128L-specific CD8^+^ T-cell response (*p* = 0.002 and *p* = 0.021, respectively). pp65-specifc CD8^+^ T-cell response was higher than gH-specific CD8^+^ T-cell response (*p* = 0.024), but no difference was observed for the other peptide pools (Figure 1E). Regarding HCMV resolution, the HCMV-specific CD8^+^ T-cell response was higher against IE-1 and pp65 than gH and gL/pUL128L (*p* < 0.001, *p* = 0.003 and *p* = 0.002, respectively) (Figure 1F).

**Figure 2:**
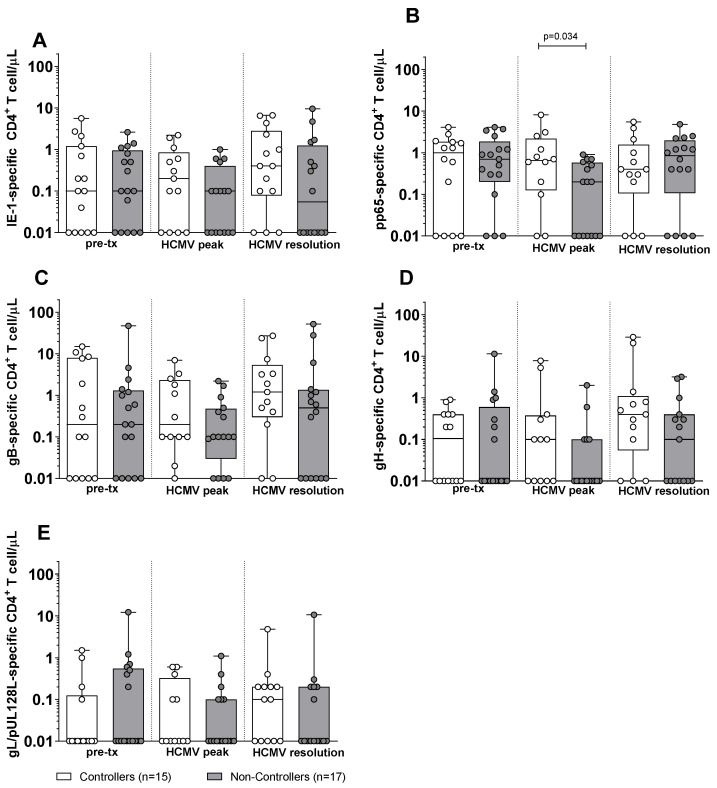
HCMV-specific CD4^+^ T-cell response against IE-1, pp65, gB, gH, and gL/pUL128L in Controllers (white bar) and Non-Controllers (grey bar) at pre-transplant (pre-tx), HCMV DNA peak, and at the resolution of HCMV infection. The HCMV-specific CD4^+^ T-cell response was quantified as the sum of all functional subsets (polyfunctional IFNγ^+^TNFα^+^IL2^+^, IFNγ^+^TNFα^+^, IFNγ^+^IL2^+^, TNFα^+^IL2^+^, and monofunctional IFNγ^+^, TNFα^+^, IL2^+^) present after stimulation. (**A**) IE-1-specific CD4^+^ T cell/μL. (**B**) pp65-specific CD4^+^ T cell/μL. (**C**) gB-specific CD4^+^ T cell/μL. (**D**) gH-specific CD4^+^ T cell/μL. (**E**) gL/pUL128L-specific CD4^+^ T cell/μL. HCMV DNA peak median time of 72 days (IQR 42–132 days after transplant) in Controllers and 52 days (IQR 38–75 days after transplant) in Non-Controllers. Resolution of HCMV infection 226 days (IQR 132–297 days after transplant) in Controllers and 206 days (IQR 141–315 days after transplant) in Non-Controllers. The analyses were perfomed using the Mann–Whitney U-test for unpaired data.

**Figure 3:**
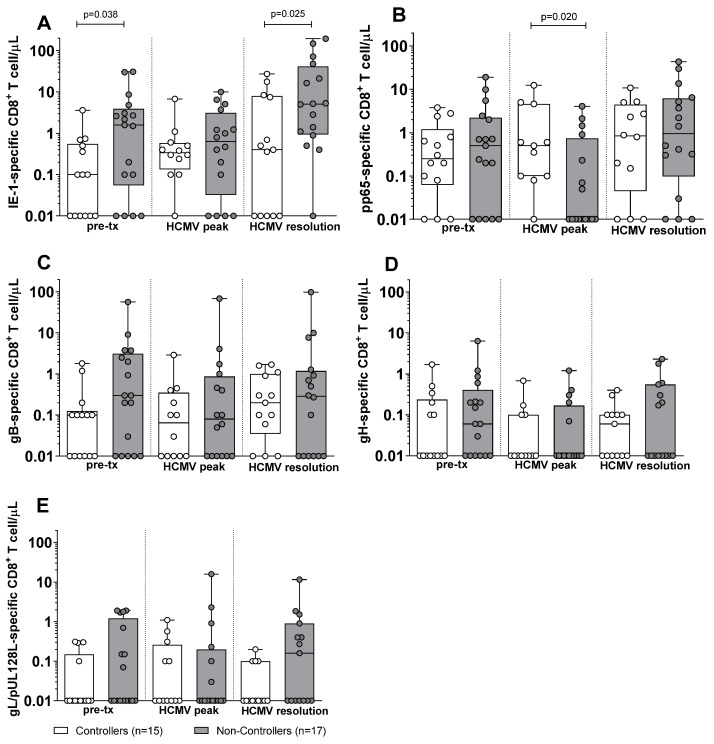
HCMV-specific CD8+ T-cell response against IE-1, pp65, gB, gH, and gL/pUL128L in Controllers (white bar) and Non-Controllers (grey bar) at pre-transplant (pre-tx), at HCMV DNA peak, and at the resolution of HCMV infection. The HCMV-specific CD8^+^ T-cell response was quantified as the sum of all functional subsets (polyfunctional IFNγ^+^TNFα^+^IL2^+^, IFNγ^+^TNFα^+^, IFN^+^IL2^+^, TNFα^+^IL2^+^, and monofunctional IFNγ^+^, TNFα^+^, IL2^+^) present after stimulation. (**A**) IE-1-specific CD8^+^ T cell/μL. (**B**) pp65-specific CD8^+^ T cell/μL. (**C**) gB-specific CD8^+^ T cell/μL. (**D**) gH-specific CD8^+^ T cell/μL. (**E**) gL/pUL128L-specific CD8^+^ T cell/μL. HCMV DNA peak median time of 72 days (IQR 42–132 days after transplant) in Controllers and 52 days (IQR 38–75 days after transplant) in Non-Controllers. Resolution of HCMV infection 226 days (IQR 132–297 days after transplant) in Controllers and 206 days (IQR 141–315 days after transplant) in Non-Controllers. The analyses were performed using the Mann–Whitney U-test for unpaired data.

Subsequently, we compared the antigen-specific T-cell response in Controllers and Non-Controllers. At pre-transplant, no difference was observed in the antigen-specific CD4^+^ T-cell response against IE-1, pp65, gB, gH, and gL/pUL128L between Controllers and Non-Controllers (Figure 2). Also, at HCMV DNA peak, no difference was observed in the IE-1, gB, gH, and gL/pUL128L-specific CD4^+^ T-cell response (Figure 2A,C–F), with the exception for the pp65-specific CD4^+^ T-cell response, which was higher in Controllers than in Non-Controllers (*p* = 0.034, Figure 2B). At the resolution of HCMV infection, no difference was observed for the antigen-specific CD4^+^ T-cell response between Controllers and Non-Controllers (Figure 2). Regarding the antigen-specific CD8^+^ T cell, Non-Controllers showed higher level of IE-1-specific CD8^+^ T-cell response (*p* = 0.038) than Controllers at pre-transplant (Figure 3A), while no difference was observed for the other HCMV peptide pools between Controllers and Non-Controllers (Figure 3). At HCMV DNA peak, the pp65-specific CD8^+^ T-cell response was higher in Controllers than Non-Controllers (*p* = 0.020, Figure 3B), while for the other HCMV peptide pool, no difference was observed (Figure 3). Instead, at resolution of HCMV infection, Non-Controllers showed higher IE-1-specific CD8^+^ T-cell response (*p* = 0.025) than Controllers (Figure 3A), while no difference was observed for other HCMV peptide pools (Figure 3).

### 3.3. Cytokine Secretion Profile of pp65-Specific CD4^+^ and CD8^+^ Mono- and Polyfunctional T Cells in Controllers and Non-Controllers

**Figure 4:**
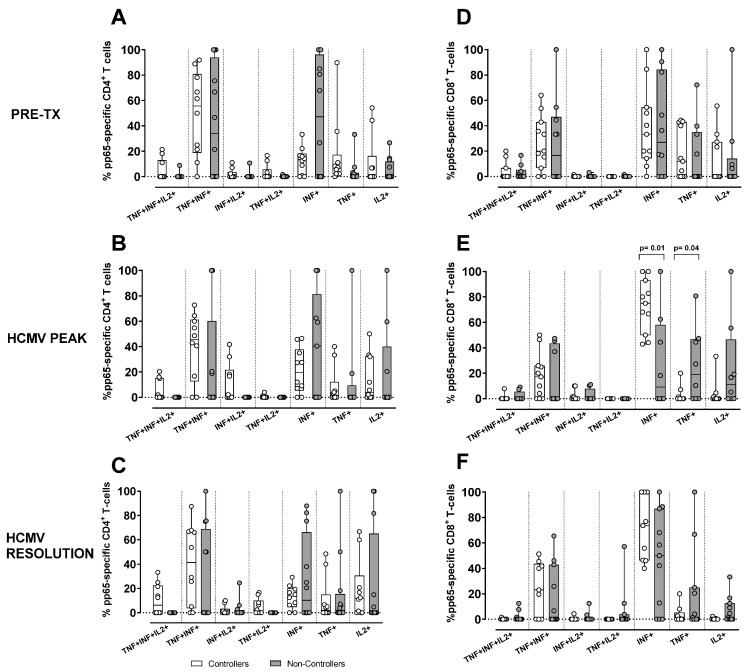
Percentage of pp65-specific CD4^+^ (**A**–**C**) and CD8^+^ (**D**–**F**). T-cell response according to the different cytokine production combinations in Controllers (white bar) and Non-Controllers (grey bar) at different time points (pre-tx, HCMV DNA peak, and resolution of the infection). The cytokine combination was trifunctional (IFNγ^+^TNFα^+^IL2^+^), bi-functional (IFNγ^+^TNFα^+^, IFNγ^+^IL2^+^ and TNFα^+^IL2^+^) and monofunctional (IFNγ^+^, TNFα^+^, and IL2^+^). (**A**) Frequency of pp65-specific CD4^+^ T-cell response at pre-tx. (**B**) Frequency of pp65-specific CD4^+^ T-cell response at HCMV DNA peak. (**C**) Frequency of pp65-specific CD4^+^ T-cell response at the resolution of HCMV infection. (**D**) Frequency of pp65-specific CD8^+^ T-cell response at pre-tx. (**E**) Frequency of pp65-specific CD8^+^ T-cell response at the HCMV DNA peak. (**F**) Frequency of pp65-specific CD8^+^ T-cell response at the resolution of HCMV infection. The analysis were perfomed using the Mann–Whitney U-test for unpaired data.

**Figure 5:**
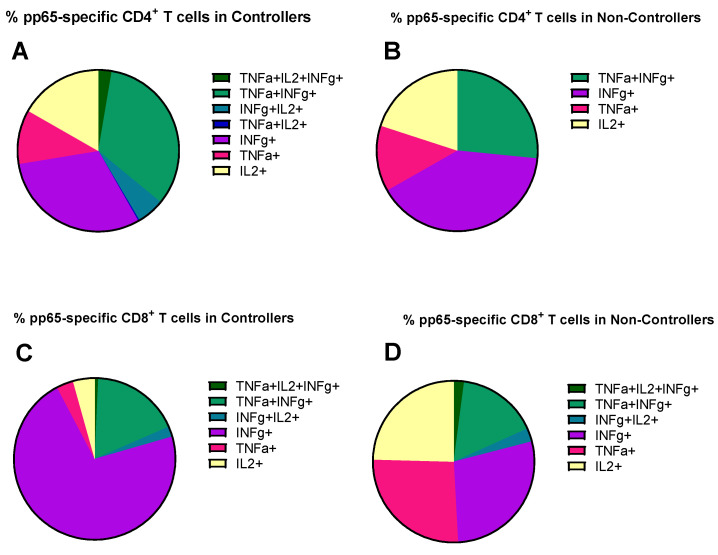
The pie chart shows the distribution of different cytokine combinations, namely trifunctional (IFNγ^+^TNFα^+^IL2^+^), bi-functional (IFNγ^+^TNFα^+^, IFNγ^+^IL2^+^, and TNFα^+^IL2^+^), and monofunctional (IFNγ^+^, TNFα^+^ and IL2^+^) in pp65-specific CD4^+^ and CD8^+^ T cells at HCMV DNA peak in Controllers (median: 61, IQR [42, 141] days after onset infection) and Non-Controllers (52, [38, 75] days after onset infection). (**A**) Mean of percentage of pp65-specific CD4^+^ T cells in Controllers; (**B**) mean percentage of pp65-specific CD4^+^ T cells in Non-Controllers; (**C**) mean of percentage of pp65-specific CD8^+^ T cells in Controllers; (**D**) mean of percentage of pp65-specific CD8^+^ T cells in Non-Controllers.

Regarding the evaluation of the antigen-specific response in terms of cytokine secretion profile, we quantified the contribution of each functional subset relative to the total number of pp65-specific T cells, defined as the sum of the seven functional subsets (trifunctional IFNγ^+^TNFα^+^IL2^+^, bi-funtional IFNγ^+^TNFα^+^, IFNγ^+^IL2^+^, TNFα^+^IL2^+^, and monofunctional IFNγ^+^, TNFα^+^, IL2^+^) in Controllers and Non-Controllers at three time points (pre-transplant, HCMV DNA peak, and resolution of HCMV infection). The analysis was performed for pp65-specific CD4^+^ and CD8^+^ T cells (Figure 4), where the total pp65-specific CD4^+^ and CD8^+^ T cell response was >0.05%. This is because this antigen specificity, as reported above, was the one resulting in a difference between Controllers and Non-Controllers at the peak of infection. In Controllers, the pp65-specific CD4^+^ T-cell response was observed across all subsets, including the trifunctional IFNγ^+^TNFα^+^IL2^+^ subset and the bi-functional IFNγ^+^IL2^+^ subset (Figure 4A–C), whereas these functional subsets were absent in Non-Controllers (Figure 4A–C). In Non-Controllers, only the monofunctional subsets IFNγ^+^, TNFα^+^, and IL2^+^, as well as the bi-functional IFNγ^+^TNFα^+^ subset, were detected (Figure 4A–C). Regarding the pp65-specific CD8^+^ T-cell response, all functional subsets were detected in both Controllers and Non-Controllers (Figure 4D–F), although the trifunctional IFNγ^+^TNFα^+^IL2^+^ subset and the bi-functional IFNγ^+^IL2^+^ subset were poorly represented (Figure 4D–F), Non-Controllers, however, showed a greater prevalence of the monofunctional TNFα^+^ and IL2^+^ subsets which were less abundant in Controllers (*p* = 0.01 and *p* = 004, respectively) at the HCMV DNA peak (Figure 4E). The pie chart illustrates the polyfunctional subsets of pp65-specific CD4^+^ and CD8^+^ T cells at the peak of HCMV DNA in Controllers and Non-Controllers (Figure 5). With the exception of IE-1, a small but detectable proportion of trifunctional (IFNγ^+^TNFα^+^IL2^+^) T cells was more frequently observed in the antigen-specific CD4^+^ T cells of Controllers than Non-Controllers (Supplementary Figures S2–S5).

## 4. Discussion

The objective of this study was to characterize HCMV-specific CD4^+^ and CD8^+^ T-cell responses, including their cytokine secretion profiles (IFNγ, TNFα, IL2), directed against multiple HCMV antigens (IE-1, pp65, gB, and the gH/gL/pUL128L complex) in solid organ transplant recipients (SOTRs) and hematopoietic stem cell transplant (HSCTR) patients experiencing HCMV reactivation.

HCMV-specific T-cell responses were analyzed at pre-transplant, during the HCMV DNA peak, and at infection resolution. The CD4^+^ T-cell response was stronger against pp65 and gB, while the CD8^+^ T-cell response was consistently higher for IE-1 and pp65. A higher pp65 CD4^+^ and CD8^+^ T-cell response at the HCMV DNA peak characterized Controllers compared to Non-Controllers. Moreover, in Controllers, CD4^+^ T cells showed a full range of functional subsets in terms of cytokine secretion profile, including trifunctional and bi-functional cells, while Non-Controllers only had monofunctional and a few bi-functional subsets. For CD8^+^ T cells, both groups had all functional subsets, but Non-Controllers had a higher number of monofunctional TNF-α^+^ and IFN-γ^+^ cells at the HCMV DNA peak.

Several HCMV-specific proteins are recognized by T cells, among which pp65 and IE-1 have consistently been identified as the main immunodominant targets of HCMV-specific T-cell responses [28,29,30]. Previous studies have indicated that protection against HCMV disease following solid organ transplantation correlates with the presence of CD8^+^ T cells producing IFNγ in response to IE-1, but not pp65 [28]. Moreover, IE-1-specific CD8^+^ T cells have been reported to exhibit lower cytotoxic potential compared with pp65-specific cells in both immunocompetent individuals and transplant recipients [31,32]. In another study, a trend toward higher IE-1-specific CD8^+^ T-cell response was observed in a cohort of liver transplant recipients who did not develop HCMV DNAemia [33]; however, this difference did not reach statistical significance [33]. In our study, we found that particular pp65-specific T cells were associated with a better control of HCMV infection. In fact, pp65-specific T cells were significantly higher at the peak of infection in patients who controlled spontaneous HCMV reactivation. On the other hand, IE-1-specific CD8^+^ T cells were significantly higher in Controllers only at pre-transplant and at the resolution of HCMV infection.

The T-cell response plays a critical role in immunocompromised patients, with CD4^+^ T cells being particularly important. Evidence indicates that immunocompromised individuals who recover both HCMV-specific CD4^+^ and CD8^+^ T cells can effectively control HCMV replication in the blood [28,34,35,36,37,38,39,40,41]. Notably, CD8^+^ T cells alone do not provide sufficient protection in the absence of a robust CD4^+^ T-cell response [8,34], and several studies have demonstrated a clear association between low HCMV-specific CD4^+^ T-cell responses and the occurrence of HCMV-related events [14]. These findings underscore the essential role of CD4^+^ T cells in coordinating effective antiviral immunity.

In our previous study, conducted in pregnant women with primary HCMV infection, we observed that antigen-specific CD4^+^ and CD8^+^ T-cell responses were elicited following stimulation with IE-1, pp65, and gB [20]. Specifically, pp65 emerged as the immunodominant antigen for CD4^+^ T cells in individuals with remote HCMV infection, whereas gB was preferentially targeted by CD8^+^ T cells in pregnant women with primary infection at early time points [20]. These findings highlight the differential hierarchy of antigen recognition, depending on both infection status and the T-cell subset involved. Data from the study of immunocompetent pregnant women and from the present study in transplant recipients underlie the prominent role of pp65-specific T cells for the control of HCMV infection in both populations. In particular, pp65-specific T cells may play a major role in preventing HCMV transmission to the fetus in seropositive women. This suggests that vaccines for the protection of either pregnant women or transplant recipients should include pp65 among antigens.

In another study, antigen specificity of T-cell responses in pregnant women with primary HCMV infection was characterized using a T-cell library approach [21]: pp65 and gB antigens were predominantly recognized by CD4^+^ T lymphocytes, whereas IE-1 and pp65 antigens were the main targets of CD8^+^ T lymphocytes. Importantly, the overall pattern of antigen-specific T-cell reactivity remained stable across both early and late phases of infection [21].

Of note, the pentameric complex gHgLpUL128L, which is a major target of the neutralizing antibody response, both in immunocompetent subjects [42,43,44,45,46] and in transplant recipients [47], does not appear to contribute significantly to the T-cell response. Data from our study suggest that monitoring T-cell response against gB, gHgLpUL128L, and also IE-1 does not provide major information regarding the ability of transplant recipients to control HCMV infection.

Regarding the polyfunctionality of HCMV-specific T-cell responses, Nebbia et al. reported that liver transplant recipients who developed HCMV DNAemia exhibited lower levels of polyfunctional HCMV-specific CD4^+^ T cells (IFNγ^+^IL2^+^) and pp65-specific CD8^+^ T cells compared with those who successfully controlled viral replication [33]. The reduction in polyfunctional CD4^+^ T cells preceded the onset of DNAemia and persisted despite ongoing viral replication, whereas lower CD8^+^ responses were observed only before DNAemia onset [33].

In our previous study, to assess the polyfunctionality of HCMV-specific CD4^+^ and CD8^+^ T-cell responses in healthy donors, we stimulated PBMCs with HCMV-infected dendritic cells, HCMV-infected cell lysate (iCL), and a 34-peptide pool (PP) [27]. Polyfunctional subsets, especially IFNγ^+^TNFα^+^IL2^+^ and IFNγ^+^TNFα^+^ T cells, were highly represented in both CD4^+^ and CD8^+^ populations, regardless of the stimulus adopted. In our cohort of immunosuppressed transplant recipients, the polyfunctional response, especially the trifunctional IFNγ^+^TNFα^+^IL2^+^ was poorly represented. Moreover, this subset was undetectable among pp65-specific CD4^+^ T cells of Non-Controllers (although the difference with Controllers was not statistically significant).

A limitation of our study is the small sample size of patients analyzed at the various time points. Nevertheless, the added value of our work lies in the detailed analysis of CD4^+^ and CD8^+^ T-cell responses to different HCMV proteins, as well as in the evaluation of T-cell polyfunctionality.

A higher pp65-specific CD8^+^ IFNγ^+^ T-cell response was observed in patients who were able to control the infection compared to those who were not. However, with respect to the pp65-specific CD4^+^ T-cell response, no difference in terms of polyfunctionality was observed between Controllers and Non-Controllers. These findings suggest that both pp65-specific stimulation and IFNγ production represent key parameters for identifying individuals capable of controlling HCMV infection, whereas the assessment of T-cell responses to other HCMV antigens or the measurement of additional cytokines did not provide further discriminative value.

In conclusion, pp65 emerges as a reliable surrogate marker of a robust T-cell immune response, whereas the pentameric complex gHgLpUL128L represents a promising candidate for eliciting an effective antibody-mediated response, rather than a T-cell-mediated response.

## Figures and Tables

**Table 1:** Patient characteristics.

Characteristics	All Patients (n = 32)	Controllers (n = 15)	Non-Controllers (n = 17)	*p* Value
Age, median [IQR]	56 [49, 64]	54 [45, 62]	57 [51, 66]	0.233
**Gender, n (%):**				
Male	20 (62)	9 (60)	11 (65)	0.999
Female	12 (38)	6 (40)	6 (35)	
**Type of transplant, n (%):**				
KTR	13 (40)	8 (53)	5 (29)	
LTR	1 (3)	0	1 (6)	
HTR	2 (6)	1 (7)	1 (6)	
HSCTR	16 (50)	6 (40)	10 (59)	

**Legend:** KTR: kidney transplant recipients; LTR: lung transplant recipients; HTR: heart transplant recipients; HSCTR: hematopoietic stem cell transplant recipients.

**Table 2:** HSCTR HLA mismatch and GvHD characteristics.

Characteristics HSCTR	All Patients (n = 16)	Controllers (n = 6)	Non-Controllers (n = 10)
**HLA mismatch, n (%)**			
haploidentical	3 (19)	0	3 (30)
mismatch	1 (6)	1 (17)	0
identical	12 (75)	5 (83)	7 (70)
**acute GvHD site, n (%)**			
skin/liver	3 (19)	0	3 (30)
skin	* 2 (12)	* 1 (17)	1 (10)
liver	1 (6)	1 (17)	0
**chronic GvHD site, n (%)**			
skin	2 (12)	0	2 (20)
liver	* 2 (12)	* 1 (17)	1 (10)
lung	1 (6)	0	1 (10)
mouth	1 (6)	0	1 (10)
**acute GvHD severity, n (%)**			
mild	* 3 (19)	* 1 (17)	2 (20)
moderate	3 (19)	1 (817)	2 (20)
**chronic GvHD severity, n (%)**			
mild	3 (19)	0	3 (30)
moderate	2 (12)	0	2 (20)
severe	* 1 (6)	* 1 (17)	0
**no GvHD, n (%)**	5 (31)	4 (67)	1 (10)

**Legend:** * One patient developed acute GvHD of the skin, followed by chronic GvHD of the liver.

## Data Availability

The data that support the findings of this study are available on request from the corresponding author. The data are not publicly available due to privacy or ethical restrictions.

## Supplementary Materials

The following supporting information can be downloaded at: https://www.mdpi.com/article/10.3390/pathogens15010053/s1, Figure S1: Cytokine Flow Cytometry (CFC) gating strategy. Figure S2: The pie chart show the distribution of different cytokine combinations tri-functional (IFNγ^+^TNFα^+^IL2^+^), bi-functional (IFNγ+TNFα^+^, IFNγ^+^IL2^+^ and TNFα^+^IL2^+^) and mono-functional (IFNγ^+^, TNFα^+^ and IL2^+^) in IE-1-specific CD4^+^ and CD8^+^ T cells at HCMV DNA peak in Controller and Non-Controllers. Figure S3: The pie chart show the distribution of different cytokine combinations tri-functional (IFNγ^+^TNFα^+^IL2^+^), bi-functional (IFNγ^+^TNFα^+^, IFNγ^+^IL2^+^ and TNFα^+^IL2^+^) and mono-functional (IFNγ^+^, TNFα^+^ and IL2^+^) in IE-1-specific CD4^+^ and CD8^+^ T cells at HCMV DNA peak in Controllers and Non-Controllers. Figure S4: The pie chart show the distribution of different cytokine combinations tri-functional (IFNγ^+^TNFα^+^IL2^+^), bi-functional (IFNγ^+^TNFα^+^, IFNγ^+^IL2^+^ and TNFα^+^IL2^+^) and mono-functional (IFNγ^+^, TNFα^+^ and IL2^+^) in IE-1-specific CD4^+^ and CD8^+^ T cells at HCMV DNA peak in Controller and Non-Controllers. Figure S5: The pie chart show the distribution of different cytokine combinations tri-functional (IFNγ^+^TNFα^+^IL2^+^), bi-functional (IFNγ^+^TNFα^+^, IFNγ^+^IL2^+^ and TNFα^+^IL2^+^) and mono-functional (IFNγ^+^, TNFα^+^ and IL2^+^) in IE-1-specific CD4^+^ and CD8^+^ T cells at HCMV DNA peak in Controllers and Non-Controllers.
