# Supplementary material for: Antigenic-Specificity and Cytokine Profile of the T-Cell Response to Human Cytomegalovirus in Transplant Recipients
Source: Pathogens. 2026 Jan 5;15(1):53. doi: 10.3390/pathogens15010053 (PMC12854486; doi:10.3390/pathogens15010053)
Supplement: Supplementary file 1 [file pathogens-15-00053-s001.zip › pathogens-4000019-supplementary.pdf]

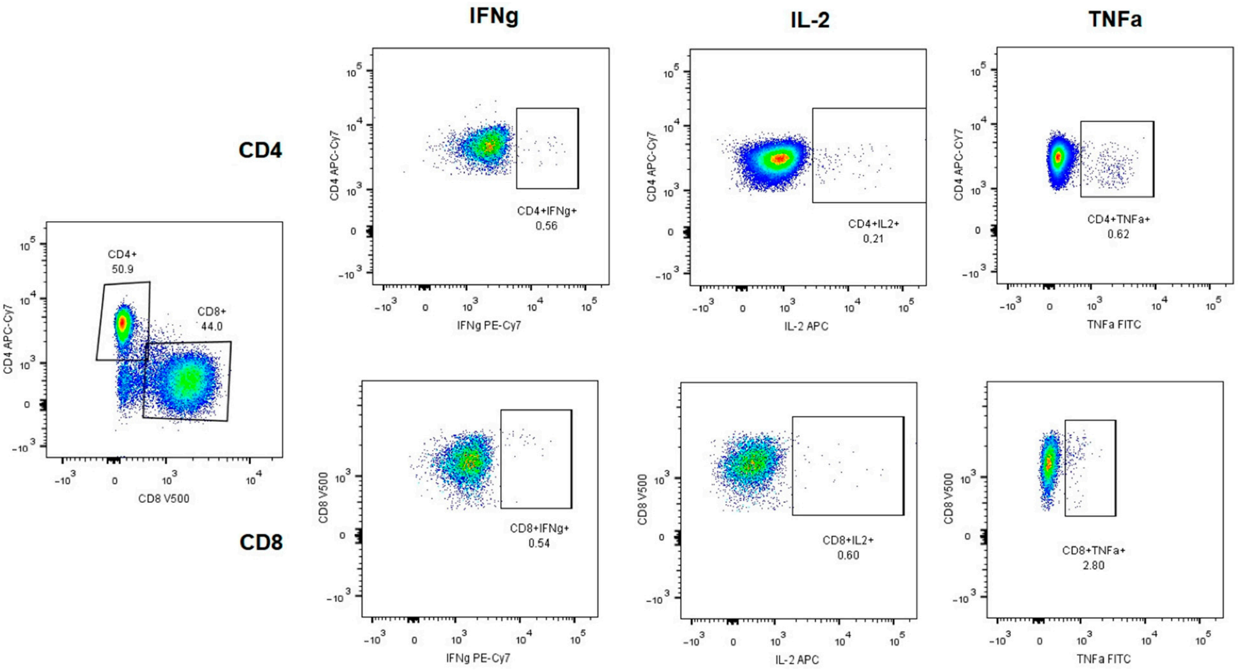

Supplementary Figure S1. Cytokine Flow Cytometry (CFC) gating strategy. Representative gating of antigen-specific $CD4^+$ and $CD8^+$ T cells from immunocompromised patients after stimulation with pp65 peptide pool. Briefly, lymphocytes cells were gated out of all events followed by live cells Pacific Blue [data not shown]. Cells were then gated as CD3 $PerCP5.5^+$ [data not shown]. T cells were further subdivided into CD4 APC $Cy7^+$ and CD8 $V500^+$ population. T cells were defined as $CD4^+$ $IFN\gamma^+$, $CD4^+$ $IL2^+$, $CD4^+$ $TNF\alpha^+$, $CD8^+$ $IFN\gamma^+$ $CD8^+$ $IL2^+$ and $CD8^+$ $TNF\alpha^+$.

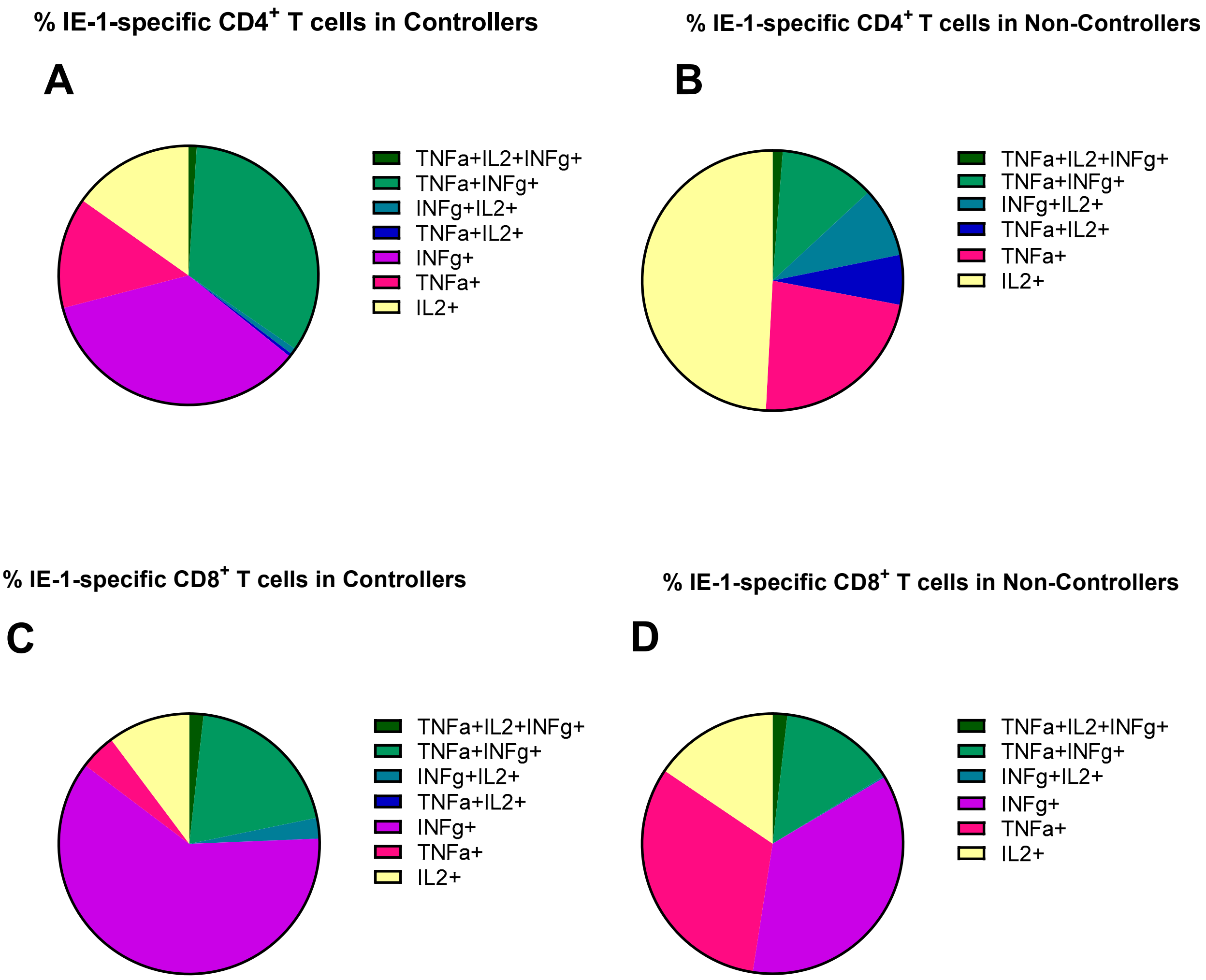

Supplementary Figure S2. The pie chart show the distribution of different cytokine combinations tri-functional ($IFN\gamma^{+}TNF\alpha^{+}IL2^{+}$), bi-functional ($IFN\gamma^{+}TNF\alpha^{+}$, $IFN\gamma^{+}IL2^{+}$ and $TNF\alpha^{+}IL2^{+}$) and mono-functional ($IFN\gamma^{+}$, $TNF\alpha^{+}$ and $IL2^{+}$) in IE-1-specific $CD4^{+}$ and $CD8^{+}$ T cells at HCMV DNA peak in Controller (median: 61, IQR [42-141] days after onset infection) and Non-Controllers (52, [38-75] days after onset infection). A) Mean of percentage of IE-1-specific $CD4^{+}$ T cells in Controllers; B) Mean percentage of IE-1-specific $CD4^{+}$ T cells in Non-Controllers; C) Mean of percentage of IE-1-specific $CD8^{+}$ T cells in Controllers; D) Mean of percentage of IE-1-specific $CD8^{+}$ T cells in Non-Controllers.

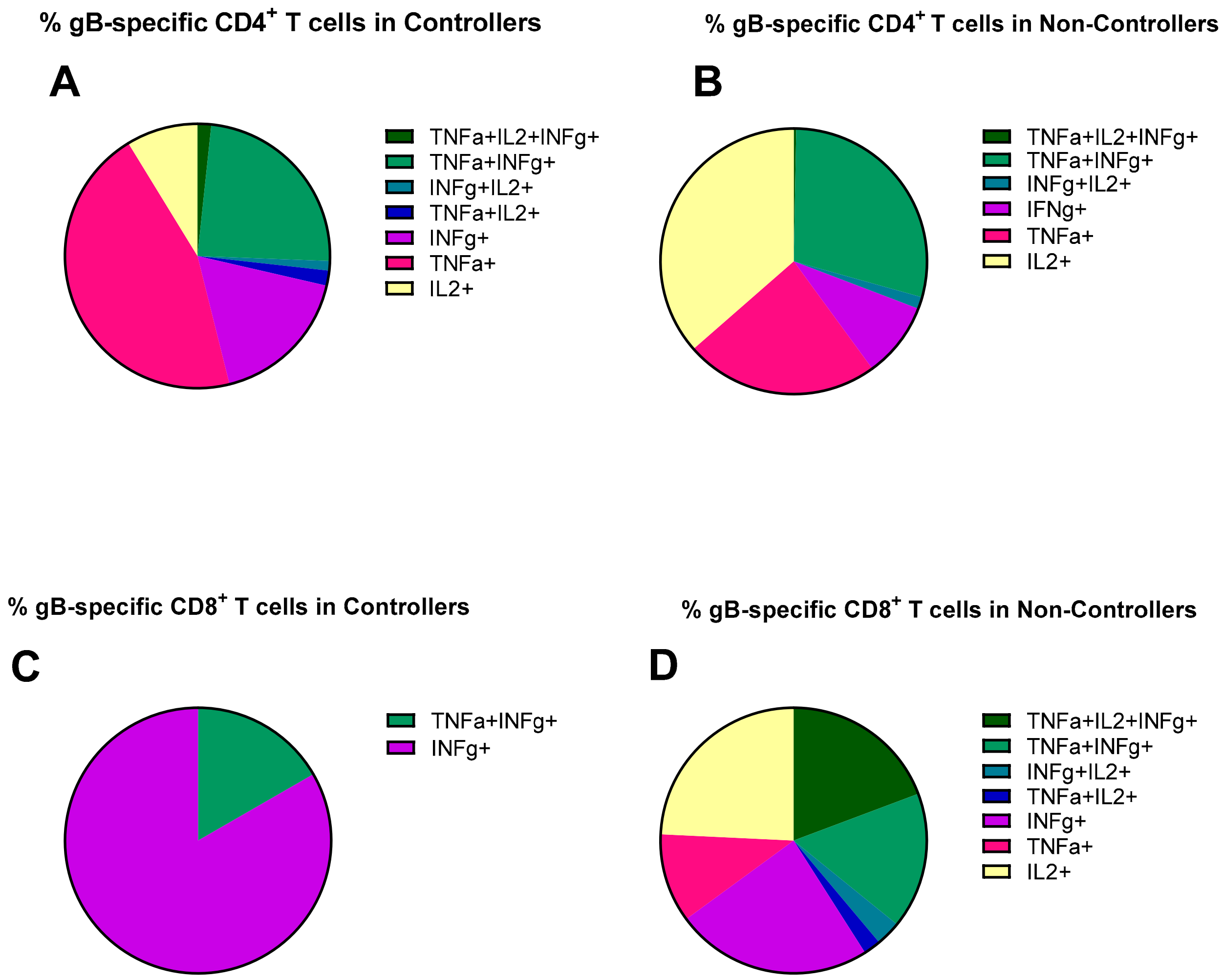

Supplementary Figure S3. The pie chart show the distribution of different cytokine combinations tri-functional ($IFN\gamma^{+}TNF\alpha^{+}IL2^{+}$), bi-functional ($IFN\gamma^{+}TNF\alpha^{+}$, $IFN\gamma^{+}IL2^{+}$ and $TNF\alpha^{+}IL2^{+}$) and mono-functional ($IFN\gamma^{+}$, $TNF\alpha^{+}$ and $IL2^{+}$) in IE-1-specific $CD4^{+}$ and $CD8^{+}$ T cells at HCMV DNA peak in Controller (median: 61, IQR [42-141] days after onset infection) and Non-Controllers (52, [38-75] days after onset infection). A) Mean of percentage of gB-specific $CD4^{+}$ T cells in Controllers; B) Mean percentage of gB-specific $CD4^{+}$ T cells in Non-Controllers; C) Mean of percentage of gB-specific $CD8^{+}$ T cells in Controllers; D) Mean of percentage of gB-specific $CD8^{+}$ T cells in Non-Controllers.

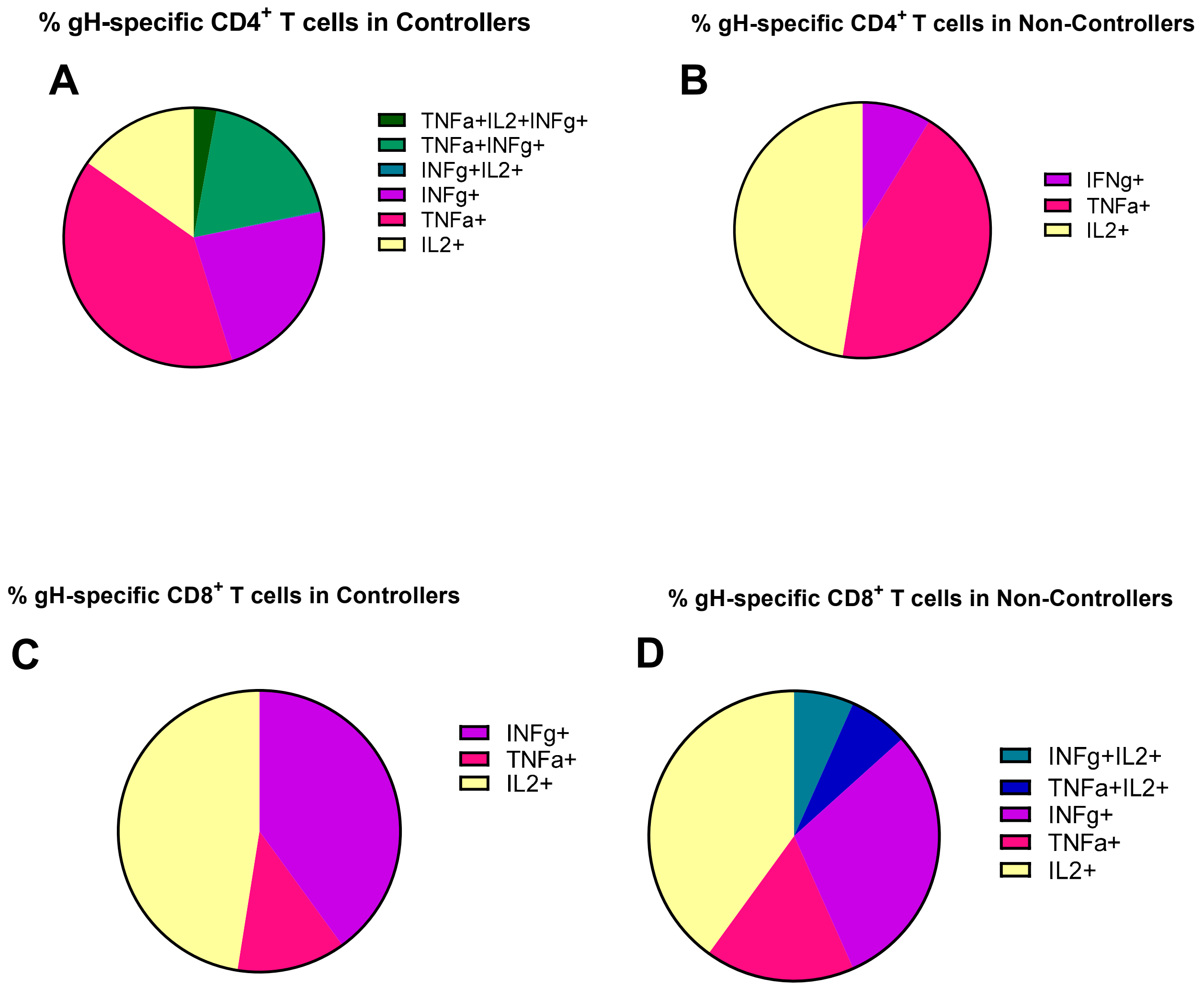

Supplementary Figure S4. The pie chart show the distribution of different cytokine combinations tri-functional (IFNγ^+TNFα^+IL2$^+$), bi-functional (IFNγ^+TNFα^+, IFNγ^+IL2$^+$ and TNFα^+IL2$^+$) and mono-functional (IFNγ^+, TNFα^+ and IL2$^+$) in IE-1-specific CD4$^+$ and CD8$^+$ T cells at HCMV DNA peak in Controller (median: 61, IQR [42-141] days after onset infection) and Non-Controllers (52, [38-75] days after onset infection). A) Mean of percentage of gH-specific CD4$^+$ T cells in Controllers; B) Mean percentage of gH-specific CD4$^+$ T cells in Non-Controllers; C) Mean of percentage of gH-specific CD8$^+$ T cells in Controllers; D) Mean of percentage of gH-specific CD8$^+$ T cells in Non-Controllers.

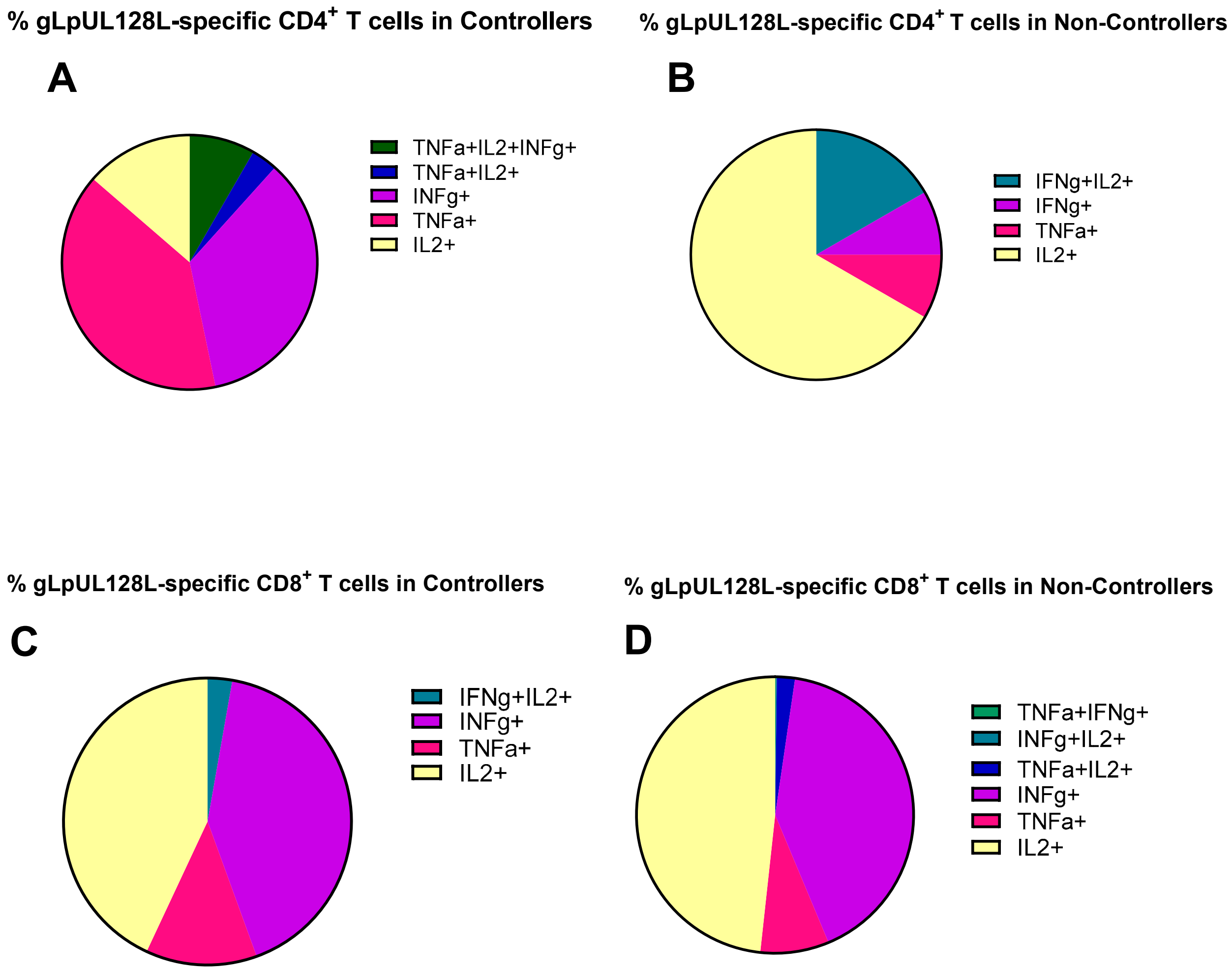

Supplementary Figure S5. The pie chart show the distribution of different cytokine combinations tri-functional ($IFN\gamma^{+}TNF\alpha^{+}IL2^{+}$), bi-functional ($IFN\gamma^{+}TNF\alpha^{+}$, $IFN\gamma^{+}IL2^{+}$ and $TNF\alpha^{+}IL2^{+}$) and mono-functional ($IFN\gamma^{+}$, $TNF\alpha^{+}$ and $IL2^{+}$) in IE-1-specific $CD4^{+}$ and $CD8^{+}$ T cells at HCMV DNA peak in Controller (median: 61, IQR [42-141] days after onset infection) and Non-Controllers (52, [38-75] days after onset infection). A) Mean of percentage of gLpUL128L-specific $CD4^{+}$ T cells in Controllers; B) Mean percentage of gLpUL128L-specific $CD4^{+}$ T cells in Non-Controllers; C) Mean of percentage of gLpUL128L-specific $CD8^{+}$ T cells in Controllers; D) Mean of percentage of gLpUL128L-specific $CD8^{+}$ T cells in Non-Controllers.
